# Editorial: Non-coding RNAs and human diseases volume 2 -long non-coding RNAs and pathogenesis of human disease

**DOI:** 10.3389/fgene.2024.1513211

**Published:** 2024-10-29

**Authors:** Shunliang Xu, Liqi Shu, Cuida Meng, Zhao-Qian Teng, Yujing Li

**Affiliations:** ^1^ Department of Neurology, The Second Hospital Cheeloo College of Medicine, Shandong University, Jinan, Shandong, China; ^2^ Rhode Island Hospital, Brown University, Providence, RI, United States; ^3^ China-Japan Union Hospital of Jilin University, Changchun, Jilin, China; ^4^ State Key Laboratory of Stem Cell and Reproductive Biology, Institute of Zoology, Chinese Academy of Sciences, Beijing, China; ^5^ Department of Human Genetics, Emory University School of Medicine, Atlanta, GA, United States

**Keywords:** long non-coding RNA, human disease, epigenetic marker, therapy, pathogenesis, transcriptome

Long non-coding RNAs (lncRNAs), a class of typically non-translatable transcripts with length of 200 nucleotides or longer, have attracted significant attention within the expansive realm of ncRNA research. Distinct from their smaller counterparts—such as microRNAs (miRNAs) and piwi-interacting RNAs (piRNAs)—lncRNAs exhibit a unique regulatory capacity, influencing critical biological processes and pathologies. Around 30,000 lncRNAs identified within human tissues exhibit remarkable tissue-specific expression patterns, suggesting their potential as biomarkers and therapeutic targets. The 2nd volume of our Research Topic, “Non-Coding RNAs and Human Diseases,” focused on the roles of lncRNAs in various human diseases, particularly on their involvement in the pathogenesis of neurological disorders, cancers, cardiac, lung, and liver diseases.

The eight original research articles and seven comprehensive review articles in this volume explored the contribution of lncRNAs to pathogenesis and translational significance. Notably, two studies centered on lung adenocarcinoma (LUAD) addressed the importance of lncRNAs in tumorigenesis. Zeng et al., elucidated the association between lncRNAs involved in endoplasmic reticulum stress (ERS) and alterations in immune landscapes, indicating that these lncRNAs could serve as promising prognostic biomarkers and potential targets for immunotherapy. In a separate study, Luo et al., characterized specific apoptosis-related lncRNAs (ApoRLs) that correlate with LUAD prognosis, underscoring their association with malignancy-associated immunomodulatory pathways.

The exploration of lncRNAs related programmed cell death (PCD) has emerged as a promising avenue for cancer therapy. Zhong et al., investigated alteration of the pyroptosis-related lncRNAs (PRlncRNAs) in acute myeloid leukemia (AML) and revealed that their risk scores could serve as indicator for prognosis, shedding the light for novel therapeutic strategies targeting PCD and the tumor microenvironment.

In the context of hepatocellular carcinoma (HCC), Wang et al., identified m7G-related lncRNAs, several them correlate with overall survival and response to chemotherapy, suggesting their potential for prognosis and therapeutic strategy. Similarly, Saklani et al. examined novel lncRNA-miRNA-mRNA networks in gallbladder cancer (GBC), exploring regulatory mechanisms and the potential for therapeutic strategies.

Dadyar et al., extended the potential of lncRNAs beyond cancers by focusing on T cell-related lncRNAs (tcr-lncRNAs) in multiple sclerosis (MS), demonstrating the contribution of tcr-lncRNAs dysregulation to disease progression and suggesting their utility as biomarkers or therapeutic targets.

In addition to research articles, seven review articles in this volume addressed current advances in lncRNAs and their functions across various diseases, four of which focused on cancer. Notable contributions include review of MIR31HG’s multifaceted role in oncogenesis by Ruan et al., and systematic review of LINC00511 in multiple functions tumorigenesis, cell invasion, metastasis, and resistance to chemotherapy by Ghafouri-Fard et al. Xiaotong et al., discussed the potential of lncRNAs as biomarker and therapeutic strategy for osteosarcoma, while Wu et al. summarized the relevance of lncRNAs in cervical cancer radiosensitivity. Another review article by Ghafouri-Fard et al., explored the interaction between ncRNAs and SIRT1, a deacetylase involved in various disorders, emphasizing the therapeutic implications of these interactions. Furthermore, Zhipu et al. summarized the role of exosomal and endogenous miRNAs in chronic rhinosinusitis with nasal polyps (CRSwNP), and Zhang et al. reviewed ncRNAs involved in ossification of the posterior longitudinal ligament (OPLL), suggesting future research directions.

In summary, this volume presents a rich tapestry of research highlighting the significance of lncRNAs as biomarkers for diagnosis and prognosis, as well as potential therapeutic targets across a spectrum of human diseases. The findings and discussions within these articles aim to provide valuable insights for researchers and clinical physicians, fostering a deeper understanding of lncRNAs in health and disease. We hope that this compilation of knowledge serves as a useful resource for advancing research and clinical applications in the field of non-coding RNAs and their significance in human health and disease.

